# Validation of a Smartphone Application for the Assessment of Dietary Compliance in an Intermittent Fasting Trial

**DOI:** 10.3390/nu14183697

**Published:** 2022-09-07

**Authors:** Isabella Baum Martinez, Beeke Peters, Julia Schwarz, Bettina Schuppelius, Nico Steckhan, Daniela A. Koppold-Liebscher, Andreas Michalsen, Olga Pivovarova-Ramich

**Affiliations:** 1 Research Group Molecular Nutritional Medicine, Department of Molecular Toxicology, German Institute of Human Nutrition Potsdam-Rehbruecke, 14558 Nuthetal, Germany; 2 Institute of Nutritional Science, University of Potsdam, 14558 Nuthetal, Germany; 3 Institute of Human Nutrition and Food Science, Faculty of Agriculture and Food Sciences, Christian-Albrecht-University Kiel, 24118 Kiel, Germany; 4 Institute of Agricultural and Nutritional Sciences, Martin Luther University Halle-Wittenberg, 06120 Halle (Saale), Germany; 5 Charité-Universitätsmedizin Berlin, Corporate Member of Freie Universität Berlin, Humboldt-Universität zu Berlin, and Berlin Institute of Health, Department of Endocrinology, Diabetes and Nutrition, Campus Benjamin Franklin, 12203 Berlin, Germany; 6 Digital Health—Connected Healthcare, Hasso Plattner Institute, University of Potsdam, 14482 Potsdam, Germany; 7 Institute of Social Medicine, Epidemiology and Health Economics, Charité-Universitätsmedizin Berlin, Corporate Member of Freie Universität Berlin, Humboldt-Universität zu Berlin, and Berlin Institute of Health, 10117 Berlin, Germany; 8 Department of Internal and Integrative Medicine, Immanuel Hospital Berlin, 14109 Berlin, Germany; 9 Department of Pediatrics, Division of Oncology and Hematology, Charité-Universitätsmedizin Berlin, Corporate Member of Freie Universität Berlin, Humboldt-Universität zu Berlin, and Berlin Institute of Health, 13353 Berlin, Germany; 10 German Center for Diabetes Research (DZD), 85764 Neuherberg, Germany

**Keywords:** dietary assessment, intermittent fasting, time-restricted eating, validation, smartphone app

## Abstract

Accurate dietary analysis of energy, nutrient intake, and meal timing in human studies using traditional dietary assessment methods (e.g., food records) is challenging and time-consuming. The widespread use of smartphones, tablets, and nutrition applications (apps) can overcome some of these problems. The objective of this study was to evaluate the validity of an FDDB smartphone app and food database compared with PRODI^®^—a professional platform for nutritional counselling using the German Nutrient Database. Dietary records were collected from 10 subjects participating in the crossover intermittent fasting trial for 2 weeks at baseline and during the eating timeframe of 8 h (early or late in the course of the day). The FDDB app and database enabled a quicker and less sophisticated analysis of food composition and timing than the PRODI^®^ software. Good agreement between the methods was found for energy and macronutrient intakes, while the FDDB data on most micronutrients and saturated/unsaturated fat intake were unreliable. In contrast to PRODI^®^, FDDB provided effective assessment of timely compliance, making it a promising tool for chrononutritional studies. Thus, the FDDB app is comparable to the traditional PRODI^®^ dietary assessment method, and can be effectively used in human dietary trials and medical practice for specific goals.

## 1. Introduction

The accurate and detailed dietary assessment in human studies using traditional dietary assessment methods (such as handwritten food records) is labor-intensive and time-consuming both for investigators and study participants. Analysis of dietary compliance—including energy and macro/micronutrient intakes, as well as meal timing—in dietary intervention trials represents an additional challenge. Furthermore, conducting chrononutritional trials investigating the effects of specific meal timings requires the assessment of timely compliance. Recent technological developments—in particular, the wide use of smartphones, tablets, and nutrition applications (apps)—can overcome some of these problems.

Smartphone apps to promote healthier lifestyles and improve health—e.g., dietary assessment apps—have developed drastically. Smartphone technology for dietary assessments demonstrating high acceptability and convenience [1,2,3] is widely used by private individuals, and is of great interest for nutritional and metabolic research. Indeed, in high-income countries, smartphone ownership rates are high. The number of smartphone users in Germany has only grown in recent years, amounting to 66.15 million smartphone owners in 2021 [4]. Statistical analysis estimated that smartphone ownership was 94.2% in the age group 14–19 years, 95.5% in the age group 20–29 years, 96% in the age group 30–39 years, and 68.2% in the age group 70+ years [5]. According to the data of the German Obesity Society (DAG), 67% of male and 53% of female Germans are overweight, and one-quarter of these people are obese. Therefore, the potential for using smartphone apps to improve the quality of dietary assessments and support weight management needs to be investigated. An app-based food diary may be more convenient and easier to use than a paper-based diary. In particular, it allows user to complete records at the instant of food intake (e.g., by a scanning a food item’s barcode, or by taking a picture). In this way, errors that may occur due to delayed recording, which is often the case when using a paper-based diary, can be avoided. Smartphone apps also enable rapid transfer of digital data from research participant to researcher. Furthermore, digital solutions allow real-time communication for monitoring the participants’ progress, resulting in an improvement in data quality and dietary compliance in nutritional research.

Further, app-based food tracking might be particularly useful in studies investigating the effects of specific meal timings (often combined with a specific food composition) on study outcomes—so-called chrononutritional studies. In particular, time-restricted eating (TRE)—an approach requiring a shortening of the daily eating window and an adherence to certain meal timings—is of great interest in the scientific community. TRE has been shown to be effective for the prevention and treatment of obesity, diabetes, and other metabolic and non-metabolic disturbances [6,7]. Currently, over 200 TRE trials are ongoing according to the ClinicalTrials.gov database (https://clinicaltrials.gov/ (accessed on 4 July 2022)). Detailed diet compliance assessment is highly important to establish a direct interrelation between trial outcomes and TRE. The potential of smartphone apps to record mealtimes has already been demonstrated by Gill and Panda [8], who developed a smartphone app to record food-intake events in real-time to analyze food patterns. The results showed that more than half of the study cohort had recording durations of approximately 15 h. Furthermore, the restriction to 10–11 h food intake was associated with weight loss [8]. However, apart from the uploading of food pictures and notes by the study participants, there was no integrated database for the detection of food composition [8]. It is becoming clear that real-time recording of food events, combined with accurate recording of food components, could provide a benefit under study conditions. In this regard, dietary apps using a food database appear to be an effective tool to simultaneously track meal timing and food composition in TRE trials. However, despite all of the abovementioned benefits, there are also some limitations when using dietary apps. One important limitation is the questionable accuracy of app-based dietary assessment tools in providing accurate information on the energy and macro/micronutrient intakes, which needs to be investigated further [3,9].

The FDDB Extender app (FDDB Internetportale GmbH, Bremen, Germany) is a smartphone dietary assessment app that is widely used by private users trying to reduce or maintain their body weight. By recording all consumed food items using barcodes or manual search in the database, along with their amounts and the timing of meals, the app provides users with information on their energy as well as macronutrient and micronutrient intakes based on the associated FDDB database. The app indicates daily required calories, which are calculated constantly based on the personal body weight goals. Additionally, the FDDB Extender app allows registration of exercise and calories burned while exercising, which are automatically included in the total summary of energy requirements. This aims at leading users to consciously follow and adapt their eating pattern to achieve their goals in a pleasant and motivating way. In addition to timely records of meal-intake events, the FDDB app also categorizes the meal type (i.e., breakfast, snack, lunch, or dinner), making it a potentially helpful tool for chrononutritional studies. However, it is currently unknown whether the FDDB app is appropriate for use in research, i.e., whether it provides accurate information on the energy and macro/micronutrient intakes comparable with professional dietary assessment software. To the best of our knowledge, to date, only one paper published in 2017 has addressed this question for the FDDB app, comparing the energy contents of 13 selected foods in the FDDB database with the German Food Database (Bundeslebensmittelschlüssel, BLS) [9]. However, no studies conducted on the FDDB validation thus far have been based on the analysis of long-term food records and addressed macro- and micronutrient intakes.

Therefore, the objective of this study was to evaluate the validity of the FDDB smartphone app and food database compared with PRODI^®^ version 6.5 (Nutri-Science GmbH, Freiburg, Germany)—a professional software platform widely used in medical practice and nutritional research in Germany. The study was conducted in the context of a trial on intermittent fasting comparing the effects of early versus late daily timeframes of food restriction.

## 2. Materials and Methods

### 2.1. Study Design

The research was conducted in the context of the ChronoFast trial in the outpatient department at the German Institute of Human Nutrition between July 2020 and December 2021. A detailed description of subject screening, inclusion and exclusion criteria, and the study design was published previously [10]. In brief, the ChronoFast trial was a 10-week randomized, controlled, crossover clinical trial performed in female subjects with overweight or obesity. The study included a four-week run-in (baseline) phase and two two-week time-restricted eating (TRE) interventions—(1) early TRE (eTRE) and (2) late TRE (lTRE), which were separated by a two-week washout period. During the intervention periods, the study subjects were instructed to consume their habitual food (and their habitual daily amounts of food) but to reduce their daily eating time window to 8 h. During eTRE, subjects were asked to consume their food between 8 a.m. and 4 p.m.; during lTRE, they were asked to eat between 1 p.m. and 9 p.m. Within the 8-hour eating window, subjects were free to divide their food intake into as many meals or snacks as they desired. Outside the eating window, participants were allowed to consume water and noncaloric drinks—e.g., tea and black coffee without sugar and milk—and limited amounts of very low-caloric diet sodas, mints, or chewing gum with sweeteners to increase compliance. During the run-in and the washout phase, participants were asked to follow their habitual eating window from before the beginning of the study. Throughout the whole study, participants were asked to avoid alcohol consumption and maintain their usual lifestyles, including physical activity and sleep timing. Taken together, the challenge of the trial was to ensure timely compliance, unchanged dietary composition, and the least possible caloric deficit.

The trial was conducted in accordance with the Helsinki Declaration of 1975. The study protocol was reviewed and approved by the Ethical Committee of the University of Potsdam, Germany (EA No. 8/2019). Written informed consent was obtained from all study subjects prior to participation. The study was registered at www.clinicaltrials.gov under the identifier NCT04351672 on 17 April 2020.

### 2.2. Study Participants and Anthropometric Measurements

The study was conducted on 10 overweight or obese non-diabetic female individuals enrolled in the ChronoFast trial. Inclusion criteria were BMI 25–35 kg/m² and age 18–70 years. All study participants were non-vegetarians and non-vegans, consumed no other special diets, and did not practice any kinds of intermittent fasting at baseline. The study subjects also did not have any eating disorders, severe intestinal diseases, history of bariatric surgery, food allergies, other diseases, or surgeries, nor did they take any medications affecting appetite, as described elsewhere [10].

The anthropometric measurements included height, weight, and body fat percentage. Body weight and height were measured using a digital scale and a wall-mounted stadiometer, respectively. Waist and hip circumferences were measured using a measuring tape. Body composition was measured using bioelectrical impedance analysis (BIA) (Akern), while fat and lean mass (kg and percentage, respectively) were calculated using the BodygramTM software version 1.3 (Akern Srl, Florence, Italy).

### 2.3. Food Intake Documentation and Nutrient Analysis

The subjects were asked to document all caloric intakes (food selection, amount, and time of each meal) for 14 consecutive days during the run-in period and during both TRE intervention phases (a total of 42 days for every individual). During the screening visit, all study subjects met a dietician, who gave them oral instructions on food documentation and usage of the app, as well as written instructions for reference during the recording period. Participants were instructed to weigh all food whenever possible and supply information on food details (e.g., brand names) and mealtimes. When weighing was not possible (e.g., when dining out), they were instructed to record the food in household measures (cups, glasses, teaspoon, etc.). Participants were explicitly asked to consistently document the meals before they sat down to eat, so that the accuracy of the registered time could be as high as possible. Study participants were able to contact the dietician if they had any questions concerning the diet or food records. Six participants documented their food intakes using paper-based handwritten dietary records, whereas four participants kept an electronic food diary using the aforementioned FDDB Extender app [9].

After collection of the food records, the study investigators analyzed them using both (i) the free FDDB food database (FDDB Internetportale GmbH, https://fddb.info/ (accessed on 4 July 2022)) and (ii) the PRODI 6.5 software (Nutri-Science GmbH, Freiburg, Germany). For analysis, the collected food intake data were incorporated in the FDDB electronic diary or PRODI^®^ software. Food records were analyzed using both FDDB and PRODI^®^ tools for total energy intake, macronutrient composition, and micronutrient composition. Additionally, the FDDB tool was used to assess meal timings and daily eating window durations to monitor participants’ adherence to their designated eating windows. To determine the daily eating window, the time interval between the first and last caloric intake of the day was calculated. In PRODI^®^, the meal timing analysis was not feasible.

### 2.4. FDDB Extender App and Food Database

The FDDB Extender app functions as a food diary. Participants log into assigned pseudonymized accounts to keep their information encrypted. By scanning the barcodes of products or by searching for them on their database, subjects can select food items and register the amounts consumed (as a self-defined portion or preset portion size), recording the time it was ingested. The application summarizes a daily food protocol stating the names of the consumed food items along with the specific time of consumption and all of the nutrients according the platform’s database. Visiting the participant’s account, the dietician is able to monitor the food intake amount, composition, and timing at any time.

The nutrient analysis in FDDB is based on the FDDB database. The FDDB platform’s database is derived from FoodData Central of the U.S. Department of Agriculture for most foods (USDA, https://fdc.nal.usda.gov/ (accessed on 4 July 2022)). In the case of German products, the nutritional item information is usually based on the Souci–Fachmann–Kraut database (available online at https://info.sfk.online/ (accessed on 4 July 2022)). For processed foods, public information from manufacturers (e.g., websites) is used as a data source, or app users are responsible for uploading the nutritional information of the products they register by scanning their barcodes. For this, app users often use the nutritional information provided in the table of nutrients on the product packaging. Although newly registered products undergo a detailed examination of macronutrients before being approved for use by other application members, micronutrients are sometimes not listed accurately. Notably, there is no requirement to state other nutrients in food labels in addition to the mandatory energy, total fat, saturated fat, total carbohydrates, sugars, protein, and salt in 100 g or 100 mL. Currently (as of July 2022), the FDDB database includes over 500,000 food items (https://fddb.info/ (accessed on 4 July 2022)).

### 2.5. PRODI^®^ Software

In parallel, all nutritional protocols were analyzed using PRODI^®^, which is a professional software platform widely used for nutritional counselling by dieticians in medical practice and nutritional research (https://www.nutri-science.de/software/prodi.php (accessed on 10 May 2021)). The database that this software utilizes is the Federal Victuals Key (BLS: Bundeslebensmittelschlüssel v. 3.02, https://blsdb.de/ (accessed on 10 May 2021)). The BLS is the standard tool used for analyzing consumption surveys in Germany. In total, 100,038 nutrients are enumerated for each of the 15,000 food items indexed. Expert literature, national and international dietary tables, and analytical values from food processing companies were used to gather the nutritional data. National nutritional tables that include approximately 1100 basic victuals were prioritized.

### 2.6. Statistical Analysis

Statistical analysis was performed using SPSS 25.0 software (SPSS, Chicago, IL, USA). Sampling distribution was analyzed using the Shapiro–Wilk test. Abnormally distributed data were log-transformed before analysis. For the comparison of two groups, the paired Student’s *t*-test or Wilcoxon test was used, depending on the distribution of the data. For the correlation analysis, Pearson’s or Spearman’s test was used, depending on the distribution of the data. A repeated-measures Bland–Altman analysis [11,12] was used to assess the relative bias (mean difference) and random error (1.96 standard deviation (SD) of the difference) between the FDDB and PRODI^®^ methods. Data are shown as means ± SD. A *p*-value < 0.05 was considered to be statistically significant.

## 3. Results

### 3.1. Participant Characteristics

The participants’ demographics and anthropometric measurements are summarized in Table 1. In total, 10 overweight non-diabetic Caucasian women participated in the study. The mean age of the participants was 61.4 ± 7.1 years, with a mean BMI of 31.4 ± 2.7 kg/m^2^, a mean waist circumference of 98.1 ± 10.6 cm, and a mean body fat percentage of 40.2 ± 7.9%. All of the study’s subjects consumed no special diets and did not practice any kinds of intermittent fasting at baseline.

### 3.2. Energy and Macronutrient Intakes at Baseline and during the TRE Periods

**Figure 1:**
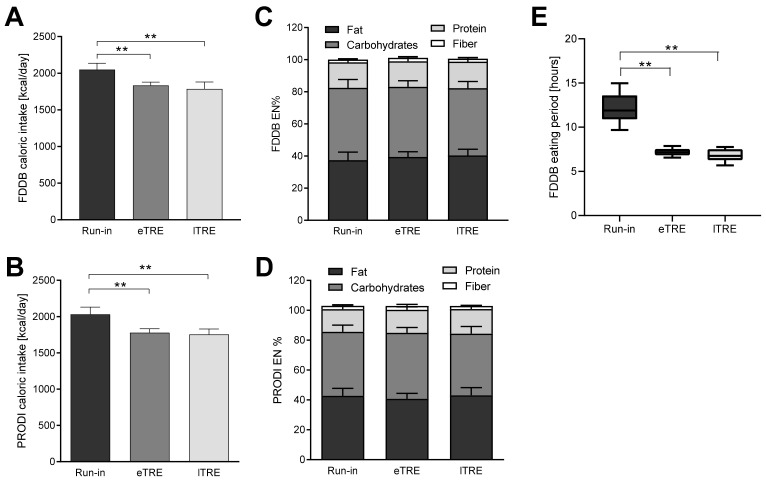
Energy and macronutrient intakes and eating time windows in the trial: Food record analysis was conducted using the FDDB and PRODI^®^ tools for energy intake (**A**,**B**), macronutrient intake (EN%, **C**,**D**), and meal timing (**E**) at baseline and during both periods of the time-restricted eating (eTRE and lTRE). EN%, percentage of total energy intake; eTRE, early time-restricted eating; lTRE, late time-restricted eating; ** *p* ≤ 0.01.

To verify dietary compliance, energy and macronutrient intakes at baseline and during both time-restricted eating phases (eTRE and lTRE) were assessed using the FDDB and PRODI databases. Both analyses showed that, despite extensive dietary advice, mean daily caloric intake was slightly lower during both interventions compared to the baseline period (FDDB: eTRE: −215 kcal/day, *p* = 0.023; lTRE: −263 kcal/day, *p* = 0.002; PRODI: eTRE: −252 kcal/day, *p* = 0.015; lTRE: −278 kcal/day, *p* = 0.005,) (Figure 1A,B. Daily macronutrient distribution remained unchanged, and was 37.4/45.1/15.8/1.8 EN% (fat/carbs/protein/fibers, respectively) at baseline, 39.4/43.7/15.9/2.0 EN% during eTRE, and 40.4/41.8/16.5/1.9 EN% during lTRE in the FDDB analysis. PRODI^®^ analysis resulted in very similar data on macronutrient distribution: 42.7/42.9/15.2/2.2 EN% (fat/carbs/protein/fiber, respectively) at baseline, 40.7/44.2/15.4/2.6 EN% during eTRE, and 43.1/41.3/16.4/2.1 EN% during lTRE (Figure 1C,D).

Analysis of the meal timing by FDDB showed that all participants successfully reduced their eating windows to under 8 h per day during both intervention phases (baseline: 12.15 ± 1.65 h; eTRE: 7.20 ± 0.44 h; lTRE: 6.84 ± 0.70 h) (Figure 1E). Timely compliance with TRE, calculated as the percentage of days when participants ate within the targeted 8 h eating window, was 95.2 ± 6.6% for eTRE and 98.5 ± 3.3% for lTRE. Analysis of the ingestion timing by FDDB is easy to perform and time-saving, because the mealtime is recorded automatically during barcode scanning or while entering consumed food items, and can later be exported in the form of an Excel document for statistical analysis. In the PRODI^®^ analysis, the automatic mealtime recording and export are not feasible, and can be performed only via the time-intensive manual processing of daily food records.

### 3.3. Comparison of the FDDB Tool with PRODI^®^ Software

**Figure 2:**
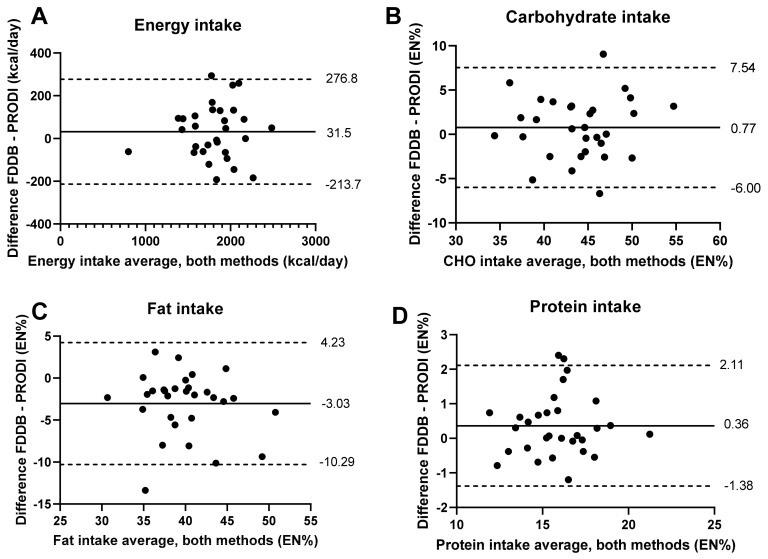
Repeated-measures Bland-Altman plots of the differences in intakes analyzed using the FDDB and PRODI tools against the mean values for the two methods for (**A**) energy, (**B**) carbohydrates, (**C**) fat, and (**D**) protein. The solid line indicates the mean difference, while the dashed lines indicate the 95% limits of agreement (LOAs) (1.96 SD) for nutrient intake. EN%, relative percentage energy intake of total macronutrient intake.

To perform inter-method comparisons, differences between the FDDB and PRODI^®^ results were calculated, and correlation analysis was conducted using the combined data of all three trial phases. There were highly significant correlations between the FDDB and PRODI^®^ tools for daily energy and macronutrient intakes (correlations ranging from 0.849 to 0.929; *p* ≤ 10^−8^) (Table 2). Macronutrients showed only minor differences for the mean percentage intakes of protein (2.3%, *p* = 0.033) and fat (7.3%, *p* = 1.07 × 10^−4^), as well as intakes in grams of protein (3.9%, *p* = 0.014), carbohydrates (3.3%, *p* = 0.031), and fat (7.3%, *p* = 1.07 × 10^−4^). In agreement with this, the Bland–Altman graphs indicated that individual differences between the two tools were within acceptable ranges (i.e., limits of agreement) for energy, carbohydrate, fat, and protein intakes, with few outliers (Figure 2).

Saturated fat and cholesterol intakes were strongly underreported with the FDDB database (−40.3%, *p* = 5.04 × 10^−10^ and −61.1%, *p* = 3.87 × 10^−15^, respectively), showing correspondingly low correlation coefficients (r = 0.532 and r = 0.625, respectively) (Table 2). For fiber, sugar, and salt, the intake differences were 10–14% between the two tools. Data on vitamins D, E, B1, B2, B6, and B12 were markedly different between the FDDB and PRODI^®^ tools (*p* < 0.05), and their correlations were lower (ranging from 0.378 to 0.745; *p* ≤ 0.05) or not significant, as shown for vitamin A. Only for vitamin C was the difference between the two tools below 10% (−8.6%, *p* = 0.017). For minerals (i.e., potassium, magnesium, calcium, iron, phosphorus, copper, zinc, chloride, iodide, manganese, sulfur), the intake was mostly underreported in the FDDB analysis, and the correlations ranged from 0.414 to 0.835 (*p* ≤ 0.05). Alcohol intake showed the highest discrepancy between the two analysis tools, being approximately 4.5-fold higher in the FDDB tool (*p* = 0.014).

## 4. Discussion

Our results showed that the FDDB app and database enabled an automatic recording of the timing and duration of the daily eating window, providing an effective assessment of timely compliance in our chrononutritional trial, whereas the meal timing analysis using the PRODI^®^ software required time-intensive manual processing of daily food records. Furthermore, FDDB showed a good agreement with the PRODI^®^ software in energy and macronutrient intake data. On the other hand, the FDDB data on most micronutrients and saturated/unsaturated fat intake were inaccurate. In sum, the FDDB app and database seem to allow a more accurate, quicker, and less sophisticated analysis of food timing than conventional approaches, with energy intake and macronutrient composition seeming reliable, making it a potentially attractive tool for nutritional and especially chrononutritional research.

In the present work, we found a good agreement between these two methods for energy intake during the run-in phase and during the eTRE and lTRE interventions, and the total energy intake values obtained in both analyses showed a high correlation coefficient. The mean individual difference was good, with −31.5 kcal (LOA −277 kcal, 214 kcal) and only one outlier. This finding is in contrast to the data of Holzmann et al. [9], who showed a large discrepancy of up to 30.6% in the energy contents of several food items between FDDB and BLS data. This discrepancy might be explained by the fact that only 13 food items were assessed in [7], whereas our research investigated long-term dietary records (for a total of 42 days) in terms of the dietary intervention study.

Furthermore, our analysis showed a high correlation of macronutrient intakes for carbohydrates, protein, and fat, with only minor differences between the two tools, as a percentage of daily energy intake and in grams (2.3–7.3%). In agreement with this, the Bland–Altman graphs indicated that individual differences between the two methods were within acceptable ranges, in compliance with the 95% level of agreement, with a 1.96 standard deviation and few outliers. Thus, our data provided a clear statement that both dietary software platforms show almost identical data for macronutrients and energy levels when long-term nutritional diaries are analyzed. Thus, for energy content and macronutrients, FDDB provides data comparable to the established professional software platform PRODI^®^, and could be used effectively in nutritional research. In particular, the FDDB app and database were used in a recently published trial to compare energy and macronutrient intakes during 8 weeks of TRE vs. continuous energy restriction intervention and 6 weeks of follow-up in 42 overweight subjects [13]. Notably, in this study, most of the study participants stated that the documentation was not an additional stress factor in their everyday life [13]. Two dietary intervention trials—one TRE trial (NCT04351672) [10] and one plant-based nutrition trial (NCT03901183)—using the FDDB app to monitor energy and macronutrient intakes as well as meal timings, are currently ongoing.

Fiber, salt, and sugar exhibited high correlation coefficients and moderate differences of 10–14% between the two tools, showing that, for these nutrients, FDDB also provides data comparable to the professional software platform PRODI^®^. However, saturated fat and cholesterol intakes were strongly underreported with the FDDB database (−40.3% and −61.1%, respectively), showing low correlation coefficients. Moreover, alcohol intake showed 4.5-fold higher values with FDDB than with PRODI^®^. However, the subjects were asked not to drink alcohol for the whole study duration, so that the amounts of alcohol detected by FDDB (but not by PRODI^®^) would mainly come from the food (such as apple juice or similar), which might explain the high discrepancy. Furthermore, data on vitamins A, D, E, B1, B2, B6, and B12 were markedly different between the FDDB and PRODI^®^ tools. Only vitamin C demonstrated a difference between the two tools below 10%, and showed the highest correlation coefficient for the software comparison (r = 0.697). A possible reason for is that the information on vitamin C content is often explicitly listed on products, giving FDDB app users the ability to enter detailed information about the contents of ascorbic acid present in industrialized products. In contrast, listing the contents of other vitamins in processed foods is not considered to be as relevant. The same applies to minerals. Indeed, for minerals (i.e., potassium, magnesium, calcium, iron, phosphorus, copper, zinc, chloride, iodide, manganese, sulfur), the intakes were mostly underreported in FDDB compared to PRODI^®^ data. As mentioned previously, disclosure of micronutrient contents on the nutrient tables of processed foods is not mandatory. Therefore, it is likely that no registration of the correct amounts of minerals and vitamins is present on the FDDB extender smartphone application. A lot of the presented data in the FDDB database are collected by users or from other databases, making them broad but at the same time imprecise, as not all food products available on the market are labeled with complete nutritional information. In contrast, all food products registered on PRODI^®^ have been analyzed with regard to all micronutrients, and present precise data on them. Taken together, our data suggest that the FDDB app does not present reliable data on saturated fat, cholesterol, vitamin, and mineral contents; thus, the application would not be suitable for dietary assessment in trials focused on the influence of these nutrients.

In recent last years, a range of studies have evaluated the feasibility and accuracy of smartphone apps for dietary assessment, demonstrating significant interest of the scientific community in this topic. One of the first studies in this field, conducted in the UK, evaluated a smartphone app named My Meal Mate (MMM), initially developed to facilitate weight loss, by comparing a digital 7-day food diary to 24-hour dietary recalls [1]. The study showed a good agreement in energy intake between the methods (a difference of 218 kJ/d, with LOAs ranging from −2434 kJ to 2022 kJ), and found the app to be useful for the assessment of group means of carbohydrate, fat, and protein intakes. Two other studies comparing smartphone apps for dietary assessment in epidemiological research with 24-hour dietary recalls also showed reliable results on the energy and macronutrient intakes at the group level [14,15]. Similarly, two clinical trials that compared mean macronutrient data between professional software platforms for the evaluation of dietary records and electronic diet recording showed no significant deviations of the macronutrients, and high correlations between both methods [16,17]. Another study compared a tablet app for assessing dietary intakes with the measured food intake/food waste method in military personnel, and provided satisfactory results in the assessment of energy, macronutrient, and selected micronutrient intakes, whereas some micronutrients showed large deviations [18]. Notably, a smartphone image-based dietary assessment app tested among Canadian adults showed large deviations from 3-day food diaries in a range of nutrients, including total energy, protein, carbohydrates, fat, saturated fatty acids, and iron [19], suggesting that the accuracy of smartphone app data has to be validated before utilization in dietary trials.

Common advantages and limitations in using dietary apps for research purposes in general, and the FDDB app in particular, must be discussed. In nutritional and chrononutritional trials, the accurate analysis of caloric intake, food composition, and timing plays an essential role in the assessment of dietary compliance and of the physiological and metabolic changes in response to the modification of nutritional patterns. To this end, using digital food records in food-tracking apps might be more advantageous compared to traditional methods, such as paper-based diaries or 24-hour recalls. Indeed, the usage of smartphone apps such as the FDDB app helps to avoid a time-intensive transfer of handwritten protocol data into food database software by a dietician, and allows records to be completed directly during the meal. Obstacles such as poor readability of handwritten food diaries or adding food to the diary afterwards could be avoided through documentation via smartphone apps.

Furthermore, the number of food items in the database must be considered to decide on the method of dietary analysis in the clinical trial. Notably, the FDDB database contains about 531,350 food items (as of 19 July 2022), with a daily increase of hundreds of new foods, and is therefore more extensive than the PRODI^®^ database. This makes food searches easier for both the recording by study participants and the further analysis by researchers. Another advantage of using the FDDB Extender app is the opportunity to create recipes for subjects to cook at home, and of a simple down-calculation to one portion with proportional listing of the individual food items.

Even though FDDB can be used as an application for smartphones, it is also possible to log in to user accounts via computer, which makes it easy for the study staff to monitor and evaluate the nutritional data. By visiting the participant’s account, the dietician can check the amount of food intake, its composition, and its timing, and in this way monitor dietary compliance almost in “real time”. A monitoring system that runs parallel to the study helps to avoid protocol errors. If needed, participants can be contacted immediately to receive support, thereby maintaining the compliance. Notably, analysis of dietary records using the PRODI^®^ software is not possible in “real time”, but only after the collection of records, excluding the possibility of rapid feedback and food behavior correction of study participants. Moreover, the meal timing analysis in the PRODI^®^ software can be performed only via the time-intensive manual processing of daily food records. Furthermore, using the FDDB smartphone app also allows study staff to monitor possible weight fluctuations and rapidly detect possible causes, e.g., deviations in eating patterns or usual calorie intake. This is essential for both isocaloric and weight loss trials. Therefore, our findings show that the FDDB app can potentially be used for such specific aims as research monitoring and, if needed, for the quick correction of timely compliance, caloric intake, and macronutrient intake.

Using dietary apps for research purposes also has several limitations. In particular, some smartphone-app-based dietary assessment tools are based on photographic images of meals, where the manual interpretation and decoding of images remain labor-intensive, and might provide an imprecise information on the caloric intake and nutrient composition [3]. For this reason, most of the currently available dietary assessment apps are similar to conventional tools, i.e., food diaries and 24-hour recalls, representing a form of self-reporting. A well-known limitation in all self-reported dietary assessments is conscious or unconscious under-reporting, which can result in serious observation and interpretation bias [20]. Nevertheless, using smartphone apps directly during the meal might markedly reduce the under-reporting and associated misinterpretation [1,21]. Moreover, the usage of apps—especially those that show an individual overview of optimal caloric and macronutrient intake—may lead to changes in eating behavior and weight loss even at baseline, resulting in data bias. This effect has already been discussed by Gill and Panda [8], who used the app “myCircadianClock” (mCC) with photo documentation to record eating times and patterns. To avoid changes in eating behavior, uploaded food images and their timestamps were immediately deleted from the participants’ smartphones after being transmitted to a server [8]. Wilkinson et al. [22] highlighted the possible problem of changes in behavioral patterns, and referred to the difficulty of tracking consistent use of the mCC app in a chrononutritional study.

Finally, using a smartphone app for food recording could be challenging, especially for the older generation, while younger generations might benefit from it. For this reason, using an app correctly could be more difficult to learn than handwritten logging, as already reported in a previous randomized controlled trial [23]. Therefore, alternatives—e.g., handwritten documentation—should be provided. Furthermore, detailed instruction in the use of the app by a dietician must be given to each participant. In this respect, the development of instructions in the form of an online presentation might be a promising tool to train subjects in advance, which could also be effectively used under the present conditions of the COVID-19 pandemic.

We also have to mention a possible limitation of our research concerning the methodology of the meal recording. Indeed, six subjects used a paper-based protocol that was transferred in parallel into FDDB and into PRODI^®^ for subsequent analysis. In contrast, four subjects entered their diets directly into the FDDB app, which means that, for these individuals, meal records were first exported from FDDB in the form of an Excel table, and then transferred to PRODI^®^ for analysis. Notably, the main aim of our study was to compare the results of the dietary record analysis using the FDDB vs. PRODI^®^ databases; therefore, inconsistency in the meal recording would not have a strong effect on these data. The flexibility in methods of dietary recording allowed us to achieve higher compliance with the study protocols in our trial, because older subjects mostly preferred handwritten protocols. Nevertheless, as mentioned above, the use of digital protocols via app can affect the quality of food recording. Therefore, in future studies, it could be feasible to collect a paper-based diary simultaneously with a digital FDDB diary in the same subjects, and to verify in this way whether the different methods of recording meals affect the results.

Finally, due to the relatively small number of subjects and the high number of tests in this study, we must acknowledge that chance findings cannot be ruled out. From this reason, and to confirm the generalizability of our results, larger studies and replication studies in other populations are needed.

## 5. Conclusions

In this study, the FDDB smartphone app demonstrated a good agreement in the obtained energy and macronutrient intake data with values generated by the traditional PRODI^®^ dietary assessment method. Furthermore, the FDDB smartphone app allows a simultaneous monitoring of meal timing and dietary composition, representing a practical and effective tool for the rapid (or even real-time) assessment of timely caloric and macronutrient compliance in chrononutritional studies. Although FDDB data on intakes of most micronutrients and saturated/unsaturated fat were unreliable, the addition and revision of these data in the FDDB database could possibly further increase its potential for usage in human dietary trials and medical practice.

In summary, the FDDB app and database enabled a quicker and less sophisticated diet recording for study participants, as well as a more effective dietary analysis for dieticians than the PRODI^®^ software, and could be beneficially used for the assessment of energy and macronutrient intakes as well as meal timing in nutritional research.

## Figures and Tables

**Table 1:** Baseline characteristics of the study participants.

Characteristics	*n* = 10
Age (year)	61.4 ± 7.1
Gender (male/female)	0/10
Height (cm)	162 ± 7
Weight (kg)	82.7 ± 10.3
BMI (kg/m^2^)	31.4 ± 2.7
Waist circumference (cm)	98.1 ± 10.6
Hip circumference (cm)	109.8 ± 4.9
Waist-to-hip ratio	0.89 ± 0.09
Body fat (%)	40.2 ± 7.9

**Table 2:** Daily energy and nutrient intakes analyzed using the PRODI^®^ and FDDB tools.

Nutrients	PRODI	FDDB	Correlation	Difference FDDB vs. PRODI	*p*-Value *
	Mean ± SD	Mean ± SD	r (p)	Δ%	
Energy (kcal)	1796 ± 328	1827 ± 328	0.926 (2.27 × 10^−13^)	17.6	0.178
Protein (g)	66.9 ± 13.7	69.5 ± 13.3	0.921 (5.36 × 10^−13^)	3.9	0.014
Protein (EN%)	15.7 ± 2.0	16.0 ± 2.1	0.907 (5.26 × 10^−12^)	2.3	0.033
Carbohydrates (g)	185.6 ± 34.5	191.9 ± 39.8	0.929 (1.31 × 10^−13^)	3.3	0.031
Carbohydrates (EN%)	43.6 ± 4.9	44.4 ± 5.1	0.759 (1.17 × 10^−6^)	1.8	0.231
Fat (g)	80.8 ± 20.0	78.1 ± 18.0	0.849 (2.96 × 10^−9^)	−3.4	0.166
Fat (EN%)	41.4 ± 5.0	38.4 ± 4.5	0.699 (1.71 × 10^−5^)	−7.3	1.07 × 10^−4^
Saturated fat (g)	34.7 ± 9.0	20.7 ± 7.6	0.532 (0.003)	−40.3	5.04 × 10^−10^
Cholesterol (mg)	317 ± 90	124 ± 49	0.625 (2.21 × 10^−4^)	−61.1	3.87 × 10^−15^
Fiber (g)	20.7 ± 7.2	17.8 ± 6.8	0.745 (2.29 × 10^−6^)	−13.6	0.002
Alcohol (g)	0.90 ± 1.62	4.93 ± 23.69	0.527 (2.77 × 10^−3^)	448	0.014
Sugar (g)	79.0 ± 23.0	70.9 ± 22.9	0.893 (3.39 × 10^−11^)	−10.3	2.35 × 10^−4^
Salt (g)	5.74 ± 1.74	4.91 ± 1.79	0.754 (3.63 × 10^−6^)	−14.5	5.88 × 10^−4^
Vitamin A (mg)	0.75 ± 0.66	1.33 ± 2.56	0.317 (0.088)	76.0	0.805
Vitamin D (µg)	3.53 ± 3.06	1.32 ± 0.81	0.500 (0.005)	−62.6	4.07 × 10^−5^
Vitamin E (mg)	11.60 ± 3.65	6.29 ± 5.86	0.725 (5.85 × 10^−6^)	−45.8	4.14 × 10^−9^
Vitamin B1 (mg)	1.05 ± 0.29	0.60 ± 0.38	0.455 (0.012)	−42.9	2.59 × 10^−5^
Vitamin B2 (mg)	1.19 ± 0.34	0.89 ± 0.51	0.554 (0.002)	−26.1	3.45 × 10^−4^
Vitamin B6 (mg)	1.28 ± 0.39	1.50 ± 1.66	0.590 (0.001)	17.2	8.04 × 10^−2^
Vitamin B12 (µg)	4.42 ± 1.85	2.16 ± 1.33	0.378 (0.039)	−51.1	6.55 × 10^−9^
Vitamin C (mg)	90.7 ± 50.8	82.9 ± 55.7	0.697 (1.91 × 10^−5^)	−8.6	0.017
Potassium (mg)	2568 ± 735	1418 ± 652	0.835 (9.66 × 10^−9^)	−44.8	3.57 × 10^−14^
Magnesium (mg)	287 ± 79	137 ± 66	0.835 (9.66 × 10^−9^)	−52.3	1.49 × 10^−15^
Calcium (mg)	598 ± 150	360 ± 156	0.679 (3.71 × 10^−5^)	−39.8	1.68 × 10^−11^
Iron (mg)	11.84 ± 3.30	4.95 ± 2.42	0.749 (1.88 × 10^−6^)	−58.2	9.15 × 10^−16^
Phosphorus (mg)	1080 ± 239	498 ± 226	0.624 (2.28 × 10^−4^)	−53.9	9.19 × 10^−16^
Copper (mg)	1708 ± 502	1184 ± 1519	0.272 (0.146)	−30.7	5.71 × 10^−4^
Zinc (mg)	8.82 ± 2.01	3.89 ± 1.81	0.539 (2.10 × 10^−3^)	−55.9	1.73 × 10^−6^
Chloride (mg)	4989 ± 4506	1338 ± 968	0.618 (2.75 × 10^−4^)	−73.2	1.73 × 10^−6^
Fluoride (mg)	586 ± 408	1024 ±1824	0.427 (0.019)	74.8	0.237
Iodide (µg)	63.3 ± 25.9	94.9 ± 302.1	0.414 (0.023)	50.0	4.90 × 10^−4^
Manganese (mg)	4447 ± 2312	2069 ± 1600	0.711 (1.06 × 10^−5^)	−53.5	1.73 × 10^−6^
Sulfur (g)	645 ± 272	226 ± 139	0.667 (5.74 × 10^−5^)	64.9	1.73 × 10^−6^

* *p*-Value is the significance level for differences between two methods. EN%, percentage of total energy intake.
